# Commentary on clim-TIME: A paradigm shift in spatially resolved perturbation mapping of the metastatic tumor microenvironment

**DOI:** 10.1016/j.gendis.2026.102299

**Published:** 2026-06-13

**Authors:** Yang Fang, Lu Bai, Lingjie Jing, Jinkai Tong, Quanjun Yang

**Affiliations:** Department of Pharmacy, Shanghai Sixth People's Hospital Affiliated to Shanghai Jiao Tong University School of Medicine, Shanghai 200233, China

In March 2026, Wang et al reported in *Cell* CLIM-TIME (CRISPR–Laser-capture microdissection Integration Mapping of the Tumor Immune Microenvironment) as a scalable, high-throughput framework for interrogating causal links along the perturbation–niche–response axis. By integrating *in vivo* CRISPR perturbation with spatially resolved multi-omic readouts at the level of clonal metastatic lesions, Wang et al identified LOXL2 as a mechanistically defined mediator of extracellular matrix (ECM) remodeling and a combination target to improve immunotherapy efficacy.^1^

Wang et al further leveraged this platform to demonstrate that knockouts of tumor suppressor genes (TSGs) in the Hippo pathway promote immune evasion and drive an ECM-related transcriptional program. Subsequent functional validation revealed that LOXL2 inhibition reshapes collagen deposition and the myeloid-infiltrated niche, thereby sensitizing tumors to T cell-based therapies (Fig. 1). This work identifies LOXL2 as a potential combination target for improving TCR-T and CAR-T efficacy. More crucially, it establishes the first scalable platform for causally interrogating the relationships among genetic perturbations, spatial ecological states, and therapeutic outcomes. It provides a robust methodological framework for studying microenvironmental mechanisms of metastatic immunotherapy resistance within a spatially resolved context.

**Figure 1:**
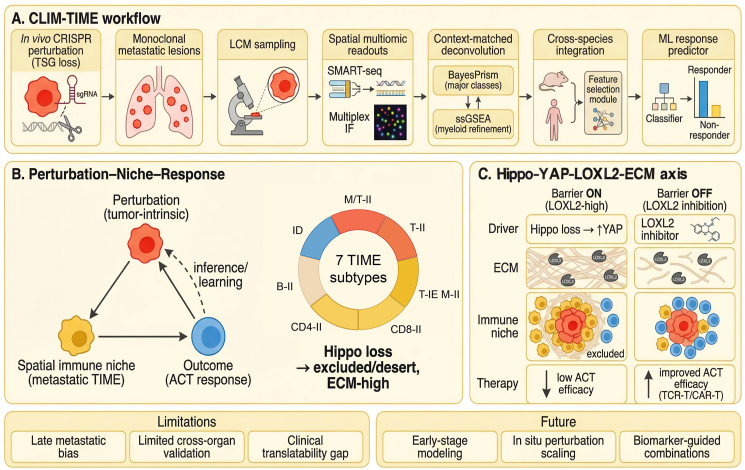
A spatially resolved conceptual framework of CLIM-TIME for causal mapping of metastatic TIME and T cell therapy response.

Spatial heterogeneity of the TIME and resistance to immunotherapy remain central challenges in oncology and have long been a focus of mechanistic studies of tumor initiation and progression. Clinically, adoptive cell therapy (ACT), particularly T cell-based modalities, has shown limited efficacy in metastatic solid tumors. A major barrier is that metastatic lesions exhibit pronounced spatial heterogeneity and spatially organized immune niches within the TIME, complicating mechanistic attribution and therapeutic stratification.^2^^,^^3^ Conventional high-throughput screens generally require tissue dissociation, which disrupts spatial architecture and weakens causal inference between tumor-intrinsic perturbations and microenvironmental states. By contrast, most spatial-omics methods are descriptive and lack scalable perturbation capacity. Consequently, the integration of perturbation screening with spatial analysis of the TIME represents a major knowledge gap.

By integrating controllable perturbations (*e.g.*, TSG loss), spatial immune niche states, and T cell therapy outcomes, CLIM-TIME provides a unified experimental framework for a genuinely spatially resolved CRISPR functional screen. This design substantially overcomes the limited causal resolution of conventional spatial-omics by closing the loop of “genetic perturbation–spatial structure–functional response” at the metastatic scale. Using monoclonal metastatic lesions as well-defined analytical units, Wang et al systematically profiled 1959 lung metastases. Importantly, the study identified seven higher-order TIME subtypes through integrated analysis combining immunofluorescence with deconvoluted SMART-seq profiles. The seven-subtype framework provides a more refined and informative representation of metastatic niche states and therefore more directly underpins the study's mechanistic and therapeutic conclusions. This high-throughput analysis established a structured mapping from TSG loss to TIME-state preference and therapeutic outcome, notably identifying that Hippo pathway loss strongly co-segregates with immune-excluded states. To robustly decode these complex microenvironments, the study relies on a context-specific computational pipeline built entirely on customized scRNA-seq references and LCM-captured SMART-seq profiles. Employing a two-step strategy, the pipeline broadly partitions major cell classes via BayesPrism before achieving high-resolution refinement of myeloid subpopulations using ssGSEA. Together, these steps transform mixed LCM transcriptomes into comparable, niche-level cellular readouts. By closely reflecting the specific TIME context, this approach mitigates inference noise from mixed signals and establishes a highly reproducible framework for expanding spatial perturbation atlases to more extensive tumor contexts.

Mechanistically, LOXL2-dependent collagen crosslinking was uncovered as a key actionable node that reinforces a matrix barrier to drive T cell exclusion. Hippo-pathway disruption activates a YAP-linked ECM remodeling program in tumor cells, with LOXL2 emerging as a key effector of collagen crosslinking and matrix organization.^4^ This remodeled ECM acts as a structural barrier that favors myeloid-rich and T cell-excluded metastatic niches. The original study further supports this model through enzymatic inactivation and rescue experiments, showing that LOXL2 catalytic activity is required for efficient matrix remodeling and immune exclusion. Accordingly, LOXL2 inhibition or depletion reshapes collagen architecture, reduces the physical constraints on T cell entry, enhances T cell infiltration, and improves the efficacy of T cell-based therapies. This mechanistic depth substantially strengthens the translational rationale for targeting the Hippo–YAP–LOXL2–ECM axis in metastatic disease.

Beyond these mechanistic insights, several considerations define the current scope of CLIM-TIME. Because the platform is presently optimized for clonal lung metastases, it is particularly well suited to resolving relatively stable TIME architectures in established metastatic lesions. In addition, although the computational pipeline enables large-scale spatial profiling, subtype-level interpretation still depends in part on inference. Accordingly, fine-grained assignments, particularly when myeloid and stromal populations display transcriptional continuity or poorly resolved boundaries, would benefit from further orthogonal validation.

More importantly, the more consequential challenges concern clinical translation. The Hippo–YAP–LOXL2–ECM axis is already supported across murine metastasis models, human xenograft systems, and retrospective clinical cohort analyses, indicating that the underlying biology is not confined to a single experimental context. Even so, its broader applicability across diverse human solid tumors, organ sites, immune landscapes, and treatment histories still requires wider validation. Furthermore, even with a biologically sound target, the development of LOXL2 as a partner for combination immunotherapy hinges on several unresolved challenges: achieving sufficient drug exposure for stromal remodeling, identifying responsive patient subsets via reliable biomarkers of immune exclusion, and optimizing treatment sequencing and safety windows in combination with T cell-based therapies.

CLIM-TIME not only enables the identification of novel microenvironmental regulators but also serves as a versatile and iterative platform for future spatial perturbation studies. By expanding to earlier stages of tumor evolution, diverse organ-specific metastases, and more *in situ* perturbation readouts, the system will gain significant functional depth. Ultimately, this framework is poised to transition from a tool for explaining resistance into a translational engine for patient stratification and rational therapy design.^5^

CLIM-TIME bridges high-throughput CRISPR screening and spatial-omics readouts by integrating genetic perturbation–spatial niche–immune response into a unified framework. It moves metastatic TIME research beyond correlative description toward attributable, comparable, and actionable causal inference. The Hippo–YAP–LOXL2–ECM axis identified through CLIM-TIME moves LOXL2 beyond a conventional therapeutic target and defines it as a mechanistically grounded candidate for combination immunotherapy. The Hippo–YAP–LOXL2–ECM axis identified through CLIM-TIME positions LOXL2 not merely as a therapeutic target but also as a mechanistically grounded candidate for combination immunotherapy. More broadly, this work reshapes the technical trajectory of TIME research and advances the field toward a perturbation-driven understanding of gene function at spatial resolution.

## CRediT authorship contribution statement

**Yang Fang:** Writing – review & editing, Writing – original draft, Validation, Methodology, Investigation, Formal analysis, Data curation. **Lu Bai:** Writing – review & editing, Writing – original draft, Validation, Methodology, Investigation, Data curation. **Lingjie Jing:** Validation, Software, Investigation, Formal analysis, Data curation. **Jinkai Tong:** Writing – review & editing, Writing – original draft, Supervision, Resources, Methodology, Funding acquisition, Conceptualization. **Quanjun Yang:** Writing – review & editing, Writing – original draft, Supervision, Project administration, Methodology, Funding acquisition, Conceptualization.

## Funding

This work was supported by the 10.13039/501100001809National Natural Science Foundation of China (No. 82272925).

## Conflict of interests

The authors declared no conflict of interests.
